# Pupillary Nystagmus as an Objective Neuro-Otological Biomarker in Vestibular Migraine: A Quantitative Pupillometric Study

**DOI:** 10.3390/audiolres16030079

**Published:** 2026-05-23

**Authors:** Augusto Pietro Casani, Nicola Ducci, Luigi Califano, Mauro Gufoni

**Affiliations:** 1 Department of Otorhinolaryngology, Pisa University Hospital, 56122 Pisa, Italy; 29550320@studenti.unipi.it (N.D.); mgufoni@gmail.com (M.G.); 2 Department of Audiology and Phoniatrics, San Pio Hospital, 82100 Benevento, Italy; luigi.califano1958@gmail.com

**Keywords:** pupillary hippus, pupillary nystagmus, vestibular migraine, vestibular examination, dizziness, episodic vertigo

## Abstract

**Background**: Vestibular migraine (VM) is a common cause of episodic vertigo, yet its diagnosis remains primarily clinical and is often complicated by the absence of reliable objective biomarkers. Pupillary nystagmus, reflecting spontaneous oscillations of pupil diameter, has been proposed as a potential clinical sign of VM, but its quantitative characterization remains limited. **Objective**: The objective of this study is to evaluate the diagnostic value of pupillary nystagmus in VM and to provide a quantitative assessment using infrared pupillometry. **Methods**: In this case–control study, 137 patients with vestibular migraine and 102 healthy controls underwent comprehensive neuro-otological evaluation, including vestibular testing and pupillometric assessment. Pupillary activity was recorded using a dedicated infrared pupillometer, and oscillatory dynamics were quantified using the Pupillary Unrest Activity Level (PUAL), which was derived through spectral analysis (Larson–Neice algorithm). Statistical comparisons were performed using non-parametric methods. **Results**: PUAL values differed significantly between VM patients and controls (Mann–Whitney test *p* < 0.001), demonstrating a clear separation between groups. A cut-off value of 0.325 was identified as the upper limit of normality, suggesting that elevated PUAL values may indicate vestibular migraine. **Conclusions**: Pupillary nystagmus represents a clinically accessible sign that can be objectively quantified through infrared pupillometry. The PUAL index provides a measurable parameter reflecting altered vestibulo–autonomic dynamics in VM and may serve as a promising neuro-otological biomarker. The integration of pupillometric analysis with clinical evaluation may improve diagnostic accuracy and support the development of objective diagnostic tools in vestibular migraine.

## 1. Introduction

Vestibular migraine (VM) is currently recognized as one of the most common causes of episodic vertigo in adults, accounting for a substantial proportion of patients presenting with recurrent vestibular symptoms in both outpatient and emergency settings [1,2,3]. Since its initial description and subsequent inclusion in the International Classification of Headache Disorders (ICHD), vestibular migraine has progressively emerged as a distinct clinical entity at the interface between neurology and neuro-otology [4,5,6]. Despite this recognition, VM remains a diagnostically challenging condition due to its heterogeneous clinical presentation and the frequent absence of pathognomonic objective findings. According to the Bárány Society and ICHD-3 criteria, the diagnosis of vestibular migraine is primarily clinical, based on the temporal association between vestibular symptoms and migrainous features together with the exclusion of alternative vestibular disorders [5,7]. However, these criteria rely heavily on patient-reported symptoms and clinical history, which may be unreliable or incomplete, particularly in patients with overlapping vestibular conditions or atypical presentations [8,9]. Consequently, diagnostic uncertainty remains common, and vestibular migraine is frequently misdiagnosed as Ménière’s disease, benign paroxysmal positional vertigo, or functional dizziness [10,11,12]. From a pathophysiological perspective, vestibular migraine is believed to arise from complex interactions between central sensory processing, vestibular pathways, and migraine-related mechanisms. These include cortical spreading depolarization, altered brainstem excitability, and dysfunctional multisensory integration [13,14,15]. Neuroimaging and neurophysiological studies have demonstrated abnormalities within vestibulo–thalamo–cortical networks, supporting the concept of VM as a disorder of central vestibular processing rather than a purely peripheral dysfunction [16,17,18]. Nevertheless, the extent to which peripheral vestibular involvement contributes to the clinical phenotype remains debated. Objective vestibular testing in patients with vestibular migraine has produced heterogeneous and often conflicting results. Conventional vestibular assessments such as caloric testing and video head impulse testing (vHIT) are frequently normal or show non-specific abnormalities, limiting their diagnostic utility [19,20]. Similarly, vestibular evoked myogenic potentials (VEMPs) and subjective visual vertical (SVV) testing may demonstrate subtle alterations, yet these findings lack sufficient sensitivity and specificity to be considered reliable biomarkers of the disease [21,22,23,24]. As a result, VM continues to be regarded largely as a diagnosis of exclusion. In recent years, increasing attention has been directed toward subtle oculomotor abnormalities observed in patients with vestibular migraine. Several studies have reported spontaneous or positional nystagmus during both ictal and interictal phases, suggesting the presence of underlying central vestibular dysfunction [25,26]. Among these signs, pupillary nystagmus, characterized by rhythmic oscillations of pupil diameter, has been proposed as a potential indicator of vestibular imbalance and brainstem autonomic instability [27]. Recent advances in pupillometry have further expanded the physiological interpretation of these phenomena. Spontaneous oscillations of pupil diameter, commonly referred to as pupillary hippus, reflect the dynamic interaction between sympathetic and parasympathetic control mechanisms and higher-order central regulatory networks. These oscillations typically occur within a frequency range between approximately 0.04 and 2 Hz and are modulated by central autonomic and arousal-related mechanisms; pupillary dynamics are widely considered to reflect activity within brainstem noradrenergic systems, including the locus coeruleus [28]. The pupil therefore represents an accessible physiological window into central autonomic and neuromodulatory processes integrating arousal, sensory processing, and cognitive control. Within this framework, pupillary oscillations may provide indirect information about the functional stability of central vestibulo–autonomic networks. Vestibular migraine has increasingly been conceptualized as a disorder involving altered interactions between vestibular nuclei, brainstem arousal systems, and cortical sensory integration pathways. Dysregulation within these circuits could therefore manifest as abnormal oscillatory dynamics within pupillomotor pathways, potentially explaining the occurrence of pupillary nystagmus in some VM patients. Parallel to these developments, advances in functional vestibular testing have led to the introduction of modified head impulse paradigms such as the functional head impulse test (fHIT). Unlike conventional vHIT, which primarily measures vestibulo-ocular reflex gain, fHIT evaluates vestibular function under cognitively demanding visual conditions and therefore probes the functional integrity of vestibulo-ocular pathways during tasks that more closely resemble real-life sensory environments [29,30]. Preliminary evidence suggests that fHIT may reveal subtle vestibular dysfunction in central vestibular disorders, including vestibular migraine, even when traditional gain-based measurements remain within normal limits [31,32,33]. Taken together, these developments suggest that integrating advanced functional vestibular testing with physiological markers derived from pupillometry may provide new insights into the neuro-otological mechanisms of vestibular migraine. Pupillary oscillations, particularly pupillary nystagmus, may represent a non-invasive indicator of instability within vestibulo–autonomic–arousal networks, complementing established vestibular tests and potentially improving diagnostic accuracy in complex clinical scenarios. The aim of the present study is therefore to provide a comprehensive clinical and instrumental analysis of patients with vestibular migraine, with particular focus on pupillary nystagmus and functional head impulse testing. By integrating conventional vestibular assessments with advanced oculomotor and functional testing, this study seeks to identify objective patterns that may enhance the diagnostic framework of vestibular migraine and improve the understanding of its underlying neuro-otological mechanisms.

## 2. Materials and Methods

This case–control study aimed to quantitatively evaluate the diagnostic performance of the clinical sign defined as “pupillary nystagmus” in distinguishing patients with vestibular migraine from non-migraine subjects. The study included 137 participants (34 males and 103 females) aged between 18 and 75 years (mean age: 48.3 years, standard deviation ≈ 15.3 years). Patients were included if they met the diagnostic criteria for vestibular migraine according to the Bárány Society. Patients with otological or neurological diseases were excluded from the study, with the exception of migraine with or without aura. A control group of healthy subjects was also included. This group consisted of 102 individuals (10 males and 92 females), aged between 18 and 76 years (mean age 40.1 years, standard deviation ≈ 14.9 years). These subjects had no history of symptoms or clinical signs attributable to migraine. Subjects with a history of otological or neurological disorders or previous head trauma were excluded from the control group.

All participants underwent a comprehensive clinical and instrumental otoneurological evaluation. Clinical assessment included detailed medical history, otoscopy, neurological examination with cerebellar testing and evaluation of cranial nerves, pure-tone audiometry, and the assessment of spontaneous, gaze-evoked, and positional nystagmus using infrared video-oculography goggles, as well as the head-shaking test. Instrumental vestibular testing included video head impulse testing (vHIT), functional video head impulse testing (fHIT), bithermal caloric testing performed according to the Fitzgerald–Hallpike protocol, with responses analyzed using Jongkees’ formulas based on the slow-phase angular velocity of nystagmus, and cervical and ocular vestibular evoked myogenic potentials (cVEMPs and oVEMPs). Patients diagnosed with vestibular migraine also underwent contrast-enhanced magnetic resonance imaging (MRI) to exclude structural disorders of the central nervous system.

Pupillary activity was evaluated using a dedicated infrared pupillometer (Neurolight), specifically designed for the assessment of pupillary oscillatory activity.

Pupillometric recordings were performed at the end of the clinical evaluation, with subjects in a resting state in a quiet environment under standardized ambient lighting conditions. Participants were instructed to maintain a steady level of attention and avoid excessive blinking or movement during acquisition. Both patients and healthy controls were assessed under identical conditions.

The device measures spontaneous fluctuations in pupil diameter under ambient light conditions and calculates the Pupillary Unrest Activity Level (PUAL) using the Larson–Neice algorithm. The Larson–Neice algorithm consists of a standardized multi-step signal processing pipeline. First, pupil diameter time-series data are acquired at a fixed sampling frequency optimized for Fast Fourier Transform (FFT) analysis. Second, blink-related artifacts and invalid samples are corrected by replacing missing values with the last valid sample, thereby minimizing non-physiological spectral distortions. Third, slow baseline drifts related to gradual tonic pupil dilation or constriction are attenuated using low-frequency Gaussian filtering in order to isolate oscillatory activity within the frequency range of interest. The processed signal subsequently undergoes spectral decomposition using FFT, and the PUAL is calculated as the sum of spectral amplitudes within a defined frequency band (typically 0.2–2 Hz), corresponding to spontaneous pupillary oscillatory activity.

PUAL represents the spontaneous oscillatory activity of the pupil around its mean diameter and reflects parasympathetic tone originating from the Edinger–Westphal nucleus. Higher PUAL values correspond to greater amplitude and variability of pupillary oscillations, reflecting increased instability of autonomic pupillomotor control. Measurements were obtained under standardized illumination conditions (approximately 100 lux).

All pupillometric recordings were performed under standardized ambient lighting conditions. Participants were instructed to avoid the acute intake of substances known to affect autonomic function, including caffeine and nicotine, prior to testing. Medication use was carefully reviewed during the screening phase, and subjects taking drugs known to influence pupillary dynamics or autonomic function were excluded from the study.

The recording consisted of 512 samples acquired at 60 Hz (8.53 s), with frequency analysis performed within the 0.2–2.5 Hz band. Pupillary data were processed using dedicated software implementing the Larson–Neice method, which applies artifact correction, removal of slow drifts, and Fast Fourier Transform (FFT) analysis to quantify oscillatory activity. The magnitude of pupillary nystagmus was therefore expressed as a single numerical value (PUAL). Two datasets of PUAL values were obtained, corresponding to the vestibular migraine group and the control group, and were subsequently compared using statistical analysis.

Statistical analyses were performed using dedicated software programs: Jamovi (Version 2.3, 2024), and RStudio Integrated Development Environment for R (Posit Software, PBC, Boston, MA, USA, version 2025.09.0). A preliminary assessment of data distribution was carried out using the Kolmogorov–Smirnov and Shapiro–Wilk tests. Since one of the two distributions, namely that of the migraine patients, was not normally distributed, comparisons between groups were performed using non-parametric methods, specifically the Levene and the Mann–Whitney test. Binomial logistic regression analysis was performed. To evaluate the discriminative ability of PUAL between migraine patients and healthy controls, receiver operating characteristic (ROC) curve analysis was performed. The area under the curve (AUC) with 95% confidence intervals (CI) was calculated.

To further quantify the association between PUAL and migraine status, a binomial logistic regression model was fitted with migraine diagnosis as the dependent variable and PUAL as the predictor. Regression coefficients (β), standard errors (SE), odds ratios (OR), and 95% confidence intervals were computed. Model performance was assessed using McFadden’s pseudo-R^2^ and deviance statistics.

All statistical tests were two-tailed, and a *p*-value < 0.05 was considered statistically significant.

Given the large observed effect sizes and the total sample size (*n* = 239), no formal post hoc power analysis was performed, as the study was considered sufficiently powered.

Preliminary analyses were exploratory in nature and were not used as formal stopping criteria for recruitment. The final statistical analyses reported here were conducted on the full dataset after completion of enrollment.

## 3. Results

Conventional vestibular testing was largely unremarkable in most patients, although subtle abnormalities were observed during functional vestibular assessment, particularly with the functional Head Impulse Test (fHIT). A summary of the principal vestibular testing findings is reported in Table 1.

Continuous variables were visually inspected and tested for normality using the Shapiro–Wilk test. Pupil diameter fluctuation (PUAL) was normally distributed in the control group but deviated from normality in the migraine group, Table 2.

Between-group differences in PUAL were initially assessed using the independent samples *t*-test. However, given the violation of normality and homogeneity of variances (Levene’s test *p* < 0.05), the non-parametric Mann–Whitney U test was considered the primary inferential approach. Effect size for the Mann–Whitney test was reported as rank-biserial correlation, Table 3, Table 4 and Table 5.

To evaluate the discriminative ability of PUAL between migraine patients and healthy controls, receiver operating characteristic (ROC) curve analysis was performed. The area under the curve (AUC) with 95% confidence intervals (CI) was calculated. The optimal cut-off value was determined using Youden’s index [34], and corresponding sensitivity, specificity, positive and negative predictive values (PPV, NPV), and likelihood ratios (LR+ and LR−) were reported.

The robustness of the findings was supported by the consistency across multiple analytical approaches (non-parametric testing, ROC analysis, and logistic regression) and by the narrow confidence intervals of the estimated parameters.

**Figure 1:**
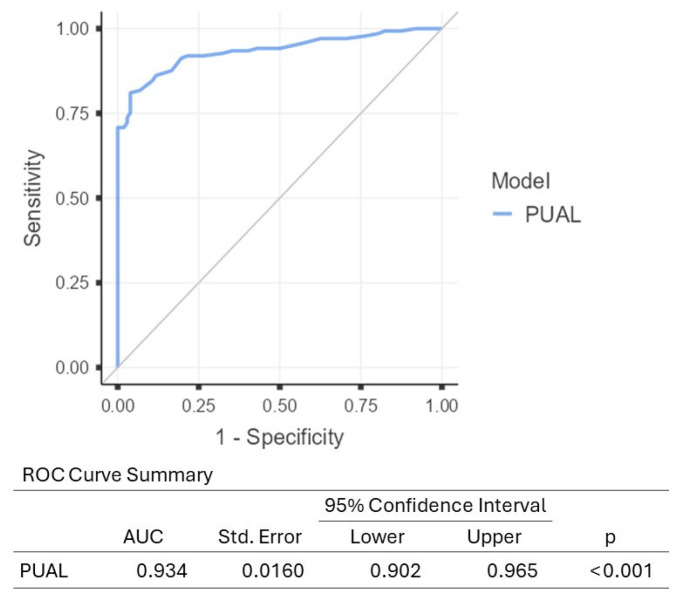
Diagnostic performance of PUAL Based on optimal cut-off (Youden’s Index). ROC curve summary: PUAL ≥ 0.325: group 1 (migraine) n = 111, group 0 (normals) n = 4; PUAL < 0.325: group 1 (migraine) n = 26, group 1 (migraine) n = 98.

**Figure 2:**
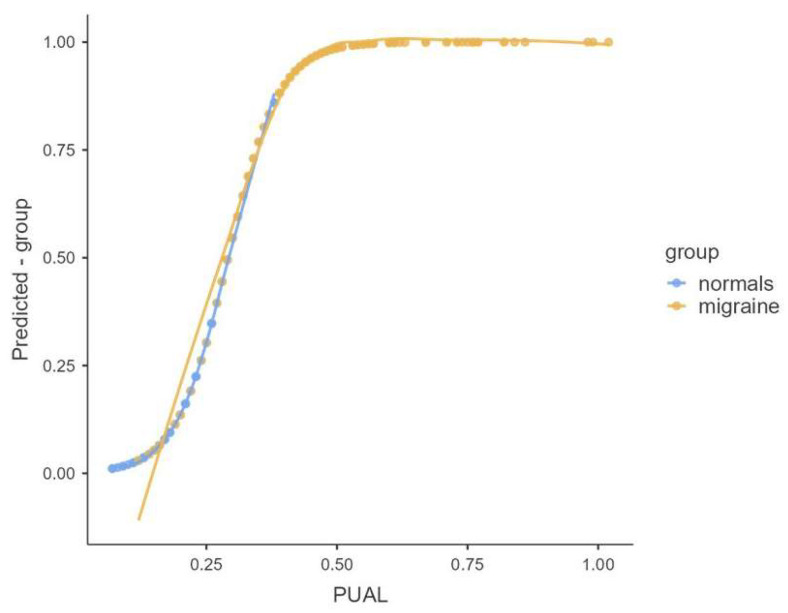
The two groups are distributed along the logistic curve with a clear separation, with a limited overlap area around the cut-off.

**Figure 3:**
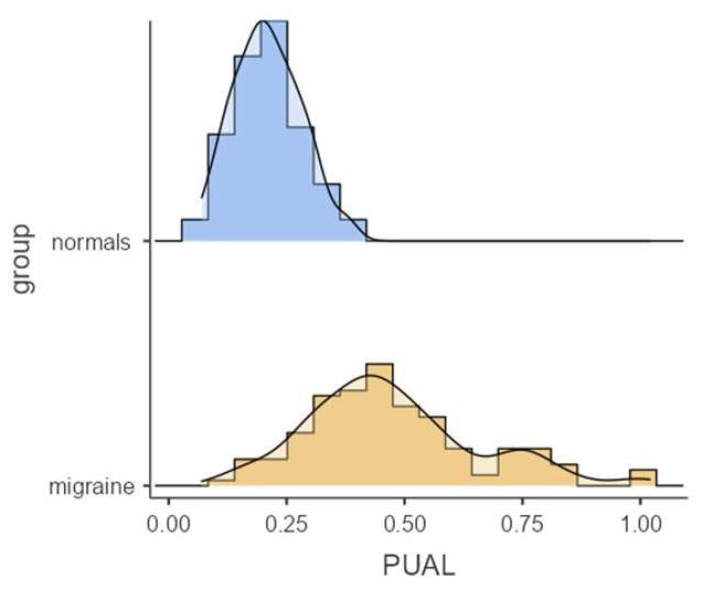
Histogram showing the distribution of PUAL values in vestibular migraine patients (yellow) and controls (blue). Control values follow a Gaussian distribution, whereas vestibular migraine patients show a shifted and non-normal distribution.

This approach yielded an upper normal limit of 0.325 (corresponding to best Youden’s index) for the PUAL index. Accordingly, PUAL values exceeding 0.325 may suggest the presence of vestibular migraine, indicating that pupillographic analysis could provide a quantitative parameter capable of distinguishing migraine patients from healthy subjects, Figure 1, Figure 2 and Figure 3.

## 4. Discussion

The diagnosis of vestibular migraine (VM) remains predominantly clinical and relies on detailed anamnesis, with diagnostic criteria requiring the coexistence of migraine features and vestibular symptoms according to the Bárány Society and ICHD-3 classification [5,7]. However, in everyday clinical practice, diagnostic uncertainty is frequent, as patients often have difficulty accurately describing their symptoms or present with overlapping vestibular disorders [8,9,10,11,12]. In this context, the identification of objective or semi-objective clinical signs becomes particularly valuable. Traditionally, VM lacks pathognomonic instrumental markers, and conventional vestibular testing often yields normal or non-specific findings [19,20,21,22,23,24]. Therefore, the search for reliable clinical indicators that may support diagnosis has long been of interest. Among these, pupillary oscillatory phenomena—commonly referred to as hippus and here interpreted as “pupillary nystagmus”—have recently gained attention. The Pupillary Unrest Activity Level (PUAL) provides a quantitative representation of spontaneous pupillary oscillations (hippus), which in the clinical setting may be perceived as pupillary nystagmus. In this context, PUAL may serve as an objective and reproducible biomarker of altered vestibulo–autonomic dynamics in vestibular migraine. Previous studies have demonstrated a high prevalence of this sign in VM patients, with reported sensitivity and specificity exceeding 90%, suggesting that its presence may represent a clinically meaningful indicator even during the interictal phase [27]. Importantly, this sign is easily detectable during bedside examination under stable lighting conditions, making it particularly attractive in routine clinical practice.

From a pathophysiological perspective, pupillary oscillations reflect the dynamic balance between sympathetic and parasympathetic activity within central autonomic networks. Altered hippus dynamics have been linked to autonomic regulation and proposed as candidate biomarkers in neurological and clinical contexts [28], while in vestibular migraine the clinical relevance of pupillary nystagmus has already been suggested by previous bedside observations [27]. However, a major limitation of pupillary nystagmus has historically been its qualitative nature, due to the absence of standardized quantitative metrics and defined thresholds of normality. In the present study, we addressed this limitation by applying infrared pupillometry to obtain continuous recordings of pupil diameter and to derive a numerical parameter, the Pupillary Unrest Activity Level (PUAL), reflecting the magnitude of pupillary oscillations. This approach enabled direct comparison between vestibular migraine patients and healthy controls and demonstrated a statistically significant separation between the two groups, thereby providing objective validation of a previously descriptive clinical sign. Notably, our findings do not negate the clinical value of bedside observation. On the contrary, pupillary oscillations are often clearly appreciable even without instrumentation, and their detection remains a rapid and accessible component of the otoneurological examination. Quantitative pupillometry should therefore be considered complementary, providing a means to standardize and validate clinical impressions, particularly in diagnostically uncertain cases.

In this context, the term “pupillary nystagmus” is used as a clinical descriptor of pupillary oscillatory activity (hippus) rather than implying a true vestibulo-ocular reflex phenomenon. Unlike ocular nystagmus, which reflects an imbalance within the vestibulo-ocular pathways, these pupillary fluctuations are thought to arise from instability within central autonomic networks, particularly at the level of the brainstem, involving the Edinger–Westphal nucleus and its modulatory inputs.

Therefore, the observed pupillary oscillations should be interpreted as a manifestation of autonomic dysregulation rather than a deficit of the vestibulo-ocular reflex. In this framework, pupillary unrest activity may represent a neuro-otological biomarker reflecting the dynamic interaction between vestibular, autonomic, and arousal systems, which are known to be altered in vestibular migraine.

These interpretations are consistent with the emerging concept of oculomics and oculometrics, which propose that eye movements and pupillary dynamics can serve as non-invasive, objective biomarkers of neurological function [35]. In this paradigm, the pupil represents a dynamic interface reflecting the activity of distributed neural networks, including vestibular, autonomic, and arousal systems. Accordingly, the presence and quantitative characterization of pupillary nystagmus in VM may be interpreted as a measurable expression of instability within vestibulo–autonomic–arousal networks rather than a purely phenomenological observation. The integration of quantitative pupillometry with clinical examination therefore represents a promising step toward bridging the gap between subjective symptom-based diagnosis and objective biomarker-based assessment. Such an approach may improve diagnostic confidence, particularly in patients who do not fully meet established criteria or present with atypical features. Nevertheless, some limitations must be acknowledged. Although our findings demonstrate a clear statistical separation between groups, larger studies are required to validate the proposed cut-off values and to assess the influence of potential confounding factors such as age, medications, and comorbidities. Furthermore, the variability of pupillary oscillations across physiological states necessitates standardized recording conditions to ensure reproducibility.

Another limitation of the present study is the use of healthy subjects as the control group. Although this approach allowed the definition of preliminary physiological reference values for PUAL, future investigations should include patients with other vestibular disorders, particularly Ménière’s disease and related episodic vertigo syndromes, in order to better assess the specificity and differential diagnostic performance of pupillometric findings in vestibular migraine.

## 5. Conclusions

Pupillary nystagmus appears to represent a reliable clinical sign supporting the diagnosis of vestibular migraine, with previous studies demonstrating high sensitivity and specificity and confirming its value even during the interictal phase [27]. Although its presence can be quantitatively assessed using dedicated instrumental methods, it retains its role as a simple and reproducible bedside test in routine clinical practice. The analysis of pupillary nystagmus through digital pupillometry emerges as a promising neuro-otological tool for the functional assessment of central vestibular disorders, particularly vestibular migraine. Preliminary findings suggest that micro-oscillations of pupil diameter—reflecting the dynamic activity of vestibulo–autonomic networks—may mirror alterations in central modulation of the vestibulo-ocular reflex and in the sympathetic–parasympathetic balance, supporting their potential role as objective biomarkers of central dysfunction [35]. The integration of qualitative clinical evaluation with quantitative pupillometric analysis may enhance diagnostic accuracy and facilitate the identification of disease-specific patterns, contributing to a more refined differential diagnosis between vestibular migraine and other causes of vertigo. Future studies based on larger cohorts and standardized protocols will be necessary to validate pupillometric parameters as reliable physiological biomarkers of vestibular migraine.

## Figures and Tables

**Table 1:** Summary of vestibular testing findings in patients with vestibular migraine.

Test	Parameter Evaluated	Main Findings
vHIT	VOR gain (horizontal and vertical canals)	Within normal limits in the majority of patients
fHIT	Percentage of correct responses during head impulses	Reduced performance in a subset of patients, suggesting functional impairment under dynamic visual conditions
Caloric test (Fitzgerald–Hallpike)	Unilateral weakness (Jongkees’ formula)	No significant canal paresis in most cases
cVEMPs	Saccular function (amplitude and latency)	Within normal limits, with no consistent asymmetry
oVEMPs	Utricular function (amplitude and latency)	Within normal limits, no pathological pattern observed
Video-oculography	Spontaneous, gaze-evoked, positional nystagmus	No persistent central or peripheral nystagmus detected outside acute episodes

**Table 2:** Descriptive statistics of PUAL in migraine patients and in healthy controls. PUAL = pupil diameter fluctuation index (dimensionless).

	Group	PUAL
N	controls	102
	migraine	137
Missing	controls	0
	migraine	0
Mean	controls	0.209
	migraine	0.474
Median	controls	0.205
	migraine	0.450
Standard deviation	controls	0.071
	migraine	0.181
IQR	controls	0.100
	migraine	0.210
Minimum	controls	0.070
	migraine	0.120
Maximum	controls	0.380
	migraine	1.020
Shapiro–Wilk W	controls	0.986
	migraine	0.962
Shapiro–Wilk p	controls	0.342
	migraine	<0.001

**Table 3:** Contingency table for PUAL at the optimal cut-off (0.325).

		Migraine	Controls	Total
PUAL	≥0.325	111	4	115
	<0.325	26	98	124
	Total	137	102	239

**Table 4:** Diagnostic accuracy of PUAL at the optimal cut-off (0.325). Predictive values depend on the observed prevalence (57.3%).

		95% Confidence Interval	
	Result	Lower	Upper
Sensitivity	81.02%	73.44%	87.21%
Specificity	96.08%	90.26%	98.92%
Positive Likelihood Ratio	20.661	7.879	54.175
Negative Likelihood Ratio	0.198	0.139	0.280
Prevalence	57.32%	50.78%	63.68%
Positive Predictive Value	96.52%	91.37%	98.64%
Negative Predictive Value	79.03%	72.68%	84.23%
Accuracy	87.45%	82.57%	91.37%

**Table 5:** Logistic regression model predicting migraine status from PUAL.

Model Coefficients Group						
Predictor	Estimate	SE	Z	*p*	Odds Ratio (OR)	95% CI for OR
Intercept	−5.920	0.788	−7.510	<0.001	_	_
PUAL	20.340	2.685	7.580	<0.001	6.83 × 10^8^	3.48 × 10^6^–1.69 × 10^11^

Note: Estimates represent the log odds of group 1 (“migraine”) vs. group 0 (“normal”).

## Data Availability

The original contributions presented in this study are included in the article. Further inquiries can be directed to the corresponding author.
